# Using computational modeling to assess decision‐making processes in a prodromal rat model of Alzheimer’s disease

**DOI:** 10.1002/alz70859_105666

**Published:** 2025-12-26

**Authors:** Mohammed U Al‐youzbaki, Salonee V Patel, Ashley L Schormans, Sarah H Hayes, Shawn N Whitehead, Brian L Allman

**Affiliations:** ^1^ Western University, London, ON Canada; ^2^ University of Rochester, Rochester, NY USA

## Abstract

**Background:**

Toward establishing behavioral biomarkers of dementia, clinical studies have shown promising results for differentiating patient groups by mathematically modeling their decision‐making processes during task performance (O’Callaghan et al., 2021; Ratcliff et al., 2022). The aim of our preclinical study was to include decision‐based modeling into the behavioral analysis of a prodromal rat model of Alzheimer’s disease (AD); an approach that we predicted would reveal genotype‐ and sex‐specific behavioral effects that were not evident in the standard assessments of task performance.

**Method:**

Male/female wildtype and transgenic Fischer 344 rats (TgAPP, which over‐express pathogenic human amyloid precursor protein, but do not spontaneously develop β‐amyloid plaques) performed a two‐alternative forced‐choice task to visually discriminate a steady versus flashing light cue. In addition to measuring performance accuracy, the rats’ reaction times were fit using an ExGauss function to assess the variability of the distribution; an important metric because increased reaction time variability is linked to age‐related cognitive decline in humans. Consistent with past studies on dementia patients, the rats’ decision‐making processes were analyzed using a drift diffusion model (DDM), which computed their rate of evidence accumulation (drift), their threshold to make decisions (bounds), as well as their initial sensory processing and final motor execution (non‐decision time).

**Result:**

Both male and female TgAPP rats performed the visual discrimination task at a modestly lower percent accuracy compared to wildtypes; however, only the female TgAPP rats showed slower reaction times, including increased variability in their timing to make decisions. Extending these genotype‐ and sex‐specific results, the DDM analysis revealed (1) slower evidence accumulation in both TgAPP sexes; (2) increased decision‐making thresholds in females compared to males, suggestive of increased response caution, and; (3) increased non‐decision times only in the TgAPP female rats.

**Conclusion:**

We show that DDM is a useful tool for revealing changes in cognitive function in a prodromal animal model of AD that were otherwise not evident using standard task metrics. Ultimately, by combining this computational approach with other biomarkers (e.g. molecular; neurophysiological), we can enhance our understanding of the earliest stages of disease progression and improve the translational potential of preclinical research.

